# Breeding short-tailed shearwaters buffer local environmental variability in south-eastern Australia by foraging in Antarctic waters

**DOI:** 10.1186/s40462-015-0044-7

**Published:** 2015-08-01

**Authors:** Maud Berlincourt, John P. Y. Arnould

**Affiliations:** School of Life and Environmental Sciences, Deakin University, Burwood, VIC 3125 Australia

**Keywords:** Procellariiforms, Movement, Foraging ecology, Reproductive performance, Geolocation, Southern Ocean

## Abstract

**Background:**

Establishing patterns of movements of free-ranging animals in marine ecosystems is crucial for a better understanding of their feeding ecology, life history traits and conservation. As central place foragers, the habitat use of nesting seabirds is heavily influenced by the resources available within their foraging range. We tested the prediction that during years with lower resource availability, short-tailed shearwaters (*Puffinus tenuirostris*) provisioning chicks should increase their foraging effort, by extending their foraging range and/or duration, both when foraging in neritic (short trips) and distant oceanic waters (long trips). Using both GPS and geolocation data-loggers, at-sea movements and habitat use were investigated over three breeding seasons (2012–14) at two colonies in southeastern Australia.

**Results:**

Most individuals performed daily short foraging trips over the study period and inter-annual variations observed in foraging parameters where mainly due to few individuals from Griffith Island, performing 2-day trips in 2014. When performing long foraging trips, this study showed that individuals from both colonies exploited similar zones in the Southern Ocean. The results of this study suggest that individuals could increase their foraging range while exploiting distant feeding zones, which could indicate that short-tailed shearwaters forage in Antarctic waters not only to maintain their body condition but may also do so to buffer against local environmental stochasticity. Lower breeding performances were associated with longer foraging trips to distant oceanic waters in 2013 and 2014 indicating they could mediate reductions in food availability around the breeding colonies by extending their foraging range in the Southern Ocean.

**Conclusions:**

This study highlights the importance of foraging flexibility as a fundamental aspect of life history in coastal/pelagic marine central place foragers living in highly variable environments and how these foraging strategies are use to buffer this variability.

## Background

In the marine environment, top predators generally rely on resources that are sparse, patchily distributed and seasonally variable [1–3]. Consequently, flexibility in their foraging behaviour in response to environmental changes is crucial, as it is relevant to breeding performances and population dynamics [4–6]. Individuals are expected to adjust their foraging effort to meet their own energy requirements and provision their offspring [7, 8]. However, the capacity to increase foraging effort is limited and species-specific. For instance, central place foragers provisioning offspring are limited in their foraging movement and duration due to the constraints of transporting food from distant feeding zones to the breeding ground and the fasting ability of their offspring [9]. Therefore, proximity of suitable feeding sites to breeding areas is essential and foraging strategies have evolved in order to maximize the foraging efficiency and the rate of net energy gain [10].

Within spatially and temporally variable environments, long-ranging central place predators such as pelagic seabirds search for highly productive habitats, changing their foraging areas and strategies depending on food availability. Consequently, they show behavioural and reproductive responses to environmental changes that impact prey availability [11, 12]. Procellariiforms, in particular, are known to show specific adaptations to the marine environment in their breeding strategy [1, 13, 14]. Unpredictability of marine environment means that adults cannot regulate food supply to the nest. As a result, accumulation of body fat and slow growth of the chicks have also been selected among many procellariiforms, enabling adults to return less frequently to the nest and chicks to survive long fasting periods [15].

During the chick-rearing period, many procellariiforms species use a dual foraging strategy including trips in different feeding areas or water masses [16, 17]. It generally involves repeated alternation between short foraging trips close to the breeding grounds and longer foraging trips extending to highly productive areas located at a great distance from the colony [18, 19]. This strategy represents a trade-off between provisioning chicks and maintaining parental body reserves. Energy transfer to chicks is more efficient after short trips but these trips generally reduce adult condition [20]. To restore their own nutritional reserves, adults forage in areas often characterized by physical structures such as frontal zones or shelf slopes with enhanced primary productivity [21–23].

Furthermore, flexibility in foraging strategy could act as a mechanism that allows adults to respond to variations in feeding grounds and adapt their foraging behaviour to buffer against variations in the distribution and abundance of resources around the breeding colonies. Inter-annual variations in resource availability could lead in the short term to a decrease in foraging success and, as a result, reduce chick provisioning and chick growth [24]. In the long term, breeding success and adult survival might also decrease [24, 25]. Therefore, despite the exploitation of distant resources, waters near the colonies could play a major role in the reproductive outcomes. The variability in movement patterns would, thus, have implications for resource allocation to survival and, ultimately, fitness [20]. Hence, local environmental variations could limit the benefits of the dual foraging strategy used by the procellariiforms.

The short-tailed shearwater (*Puffinus tenuirostris*, Temminck 1835) is a highly pelagic medium-sized (500–800 *g*) procellariiform. It is the most abundant seabird species in Australia, with approximately 23 million individuals breeding annually during the austral spring/summer (from September to April) on the many islands off the continent’s southern coast, the majority of which occur in Bass Strait [26], the shallow continental shelf area located between Tasmania and the Australian mainland. During the breeding period, adults provision their chicks on a wide range of neritic prey, especially Australian krill (*Nyctiphanes australis*), as well as myctophid fish and cephalopods, caught during short foraging trips. They also alternate these short trips with long foraging trips extensively in sub-Antarctic and Antarctic waters to restore their own body condition [27–32]. Despite numerous studies describing the general use of the Southern Ocean by the short-tailed shearwaters [33, 34], little is known of how individuals respond behaviourally to inter-annual environmental variability [35].

**Fig. 1:**
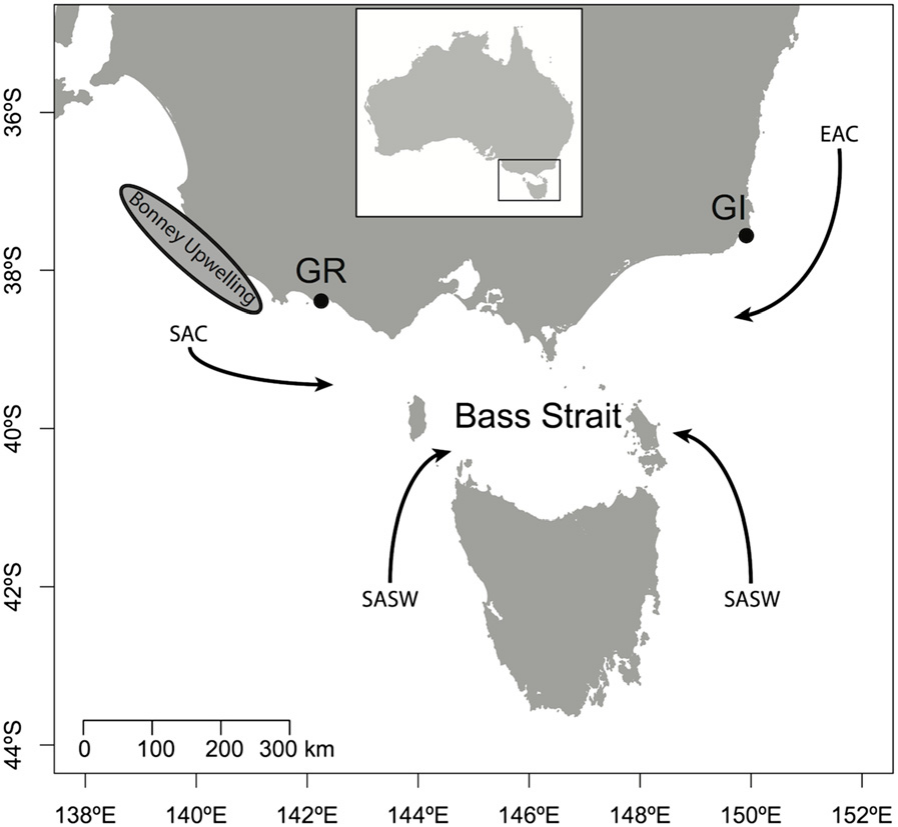
Simplified representation of water masses in south-eastern Australia (Bass Strait region) and location of breeding colonies. *EAC*: East Australian Current; *SASW*: Sub-Antarctic Surface Water; *SAC*: South Australian Current (From [36]) and Bonney Upwelling. Gabo Island (*GI*) and Griffith Island (*GR*) breeding colonies are located on the map (*closed circles*). *Inset* map shows the area’s position in relation to Australia

The region of Bass Strait is mainly influenced by the South Australian Current (SAC), the East Australian Current (EAC) and the sub-Antarctic Surface Water (SASW) [36] (Fig. 1). The warm SAC and EAC have low nutrient levels while the cold nutrient-rich SASW supports high biological productivity [37]. In addition, the Bonney Upwelling, the largest and most predictable upwelling in south-eastern Australia, provides a highly productive feeding ground for a variety of species (e.g. seabirds, fishes, whales and fur seals) [38–40]. Therefore, south-eastern Australia is marked by contrasting oceanic conditions that might influence the foraging decisions of breeding short-tailed shearwaters in response to environmental variability.

Consequently, during years with poor conditions (i.e. lower food availability), it is hypothesised that short-tailed shearwaters should increase their foraging effort, both during short and long foraging trips. As a result, individuals should extend their foraging ranges in search of profitable prey patches in order to meet both the nutritional needs of their offspring and their own. Therefore the aims of the present study were to investigate in this species: 1) how climate variability influence the foraging strategy of individuals foraging in neritic (short trips) and distant oceanic waters (long trips); and 2) its consequence on reproductive performance. As it is unlikely that substantial variation in environmental conditions, and how animal adapt to them, may be detected at a single location within the short time-frames of this study [11], short-tailed shearwater populations experiencing divergent oceanographic conditions at both extremities of Bass Strait were examined.

## Methods

### Study sites and animal handling procedures

The study was conducted at Gabo Island Lighthouse Reserve (37°33′S, 149°54′E) and Griffith Island, Port Fairy (38°22′S, 142°13′E) (Fig. 1), with breeding populations estimated at more than 6000 [41] and 15,000 breeding pairs [42], respectively. Data were collected during the chick-rearing period over three consecutive breeding seasons (2012–2014). Burrows containing hatched chicks were monitored daily in order to identify adults’ attendance patterns. Each night, chicks were weighed using a spring balance (±5 *g*). Chicks were considered to have received a meal when their mass increased between weighing, whereas mass loss indicated no feeding event (see [31]). Both adults were considered to be performing a long trip when their chicks lost weight over five consecutive days. These burrows were targeted for GPS data logger deployment because the adults were likely to perform a short trip upon return. A one-way wooden trapdoor was fitted at the entrance of the burrow. Returning parents tripped a stick that closed the trapdoor when entering the burrow. Adults were left for ~30–40 min in the burrow to feed the chick and then captured for instrumentation .

Body mass and morphometric measurements (bill length and depth, wing length) were taken using a spring balance (±5 *g*) and Vernier callipers (±1 mm), respectively. All individuals were then instrumented during early chick-rearing with an IgotU GT-120 GPS data logger (Mobile Action Technology, Taiwan) packaged in heat-shrink tubing, programmed to sample location every 5 min (i.e. to ensure locations could be recorded over a 4-day period [31]). Devices were attached with black waterproof Tesa tape to feathers on the dorsal midline (total weight was <12 *g* which is below the 3 % limit recommended for flying birds [43]). Procedures lasted <10 min and individuals were then returned to the nest to resume normal behaviour. Birds were recaptured in their burrows and devices removed after one foraging trip to sea. Upon recapture, individuals were equipped with a geolocator attached to a plastic band placed around birds’ tarsus (LAT2900, Lotek Wireless Inc, 2.5 *g* or Mk3005 (developed by the British Antarctic Survey), Biotrack, 2.5 *g*). Geolocators were retrieved during the chick-rearing period (mid- to late-March).

A total of 50, 70 and 81 nests at Gabo Island and 106, 109 and 97 at Griffith Island were marked in study plots at the time of egg-laying each year in November (2011–2013) and monitored throughout the breeding season to record the chronology of laying, hatching and fledging (and their respective success). Out of these, a total 23, 9 and 22 individuals at Gabo Island and 18, 10 and 20 individuals at Griffith Island were instrumented with both GPS and geolocator data-loggers in 2012, 2013 and 2014, respectively, once their egg have hatched. Nests of instrumented birds were monitored after deployments and their success was compared with a *Chi*-square test to the one of the remaining nests (control group) in the studied areas. Success was not significantly different between both groups throughout the study period (Gabo Island, 2012: *χ*^*2*^ = 0.43, *P* = 0.51; 2013: *χ*^*2*^ = 2.01, *P* = 0.16; 2014: *χ*^*2*^ = 1.25, *P* = 0.26 and Griffith Island, 2012: *χ*^*2*^ = 0.39, *P* = 0.53; 2013: *χ*^*2*^ = 0.87, *P* = 0.35; 2014: *χ*^*2*^ = 0.001, *P* = 0.97).

The present study was conducted following the ethical guidelines of the Deakin University Animal Ethics Committee (Approval A61-2010) and in accordance with the regulations of Department of Sustainability and Environment (Victoria, Australia, Permit 10005531).

### Oceanographic conditions

Local environmental variables were characterized in the marine habitats surrounding the breeding colonies and investigated during chick rearing (January–April) for each study period (2012–2014). A spatial grid based on the geographic limits of the tracking data (Gabo Island: from 149°19′E to 150°15′E and from 36°45′S to 37°52′S; Griffith Island: from 140°23′E to 144°34′E and from 38°20′S to 40°31′S) was built. Local ocean climate conditions were characterized by deriving weekly sea surface temperature (SST) and chlorophyll-*a* concentration (used as a proxy of primary productivity) within the studied locations. Weekly SST (°C) (AVHRR GAC SST 11 km 8-day composite) and Chl-*a* (mg · m^−3^) (MODIS Aqua 2.5 km chla 8-day composite) values were extracted from BloomWatch (http://coastwatch.pfel.noaa.gov/coastwatch/CWBrowserWW360.jsp) using the Xtractomatic routine (http://coastwatch.pfel.noaa.gov/xtracto/). Additionally, wind speed (m · s^−1^) and wind direction (°) were used to characterize conditions experienced during foraging flight. Values were obtained from Buoyweather (http://www.buoyweather.com/index2.jsp) at two locations in the proximity of the breeding colonies (Gabo Island: 150°E–38°S; Griffith Island: 143°E–39°S) as 3 hourly readings and converted to weekly means.

On a meso-scale, the strength of the Bonney Upwelling (Fig. 1) was characterized by extracting weekly SST (°C) composites during the austral summer (from January to March 2012–14) within a zone between 36°S and 38.5°S along the southeastern coast of mainland Australia to the 1000 m isobath (i.e. edge of the continental plateau) [44]. On a larger scale, the Southern Oscillation Index (SOI) was used as a proxy of macro-scale oceanographic process. El Niño Southern Oscillation (ENSO) is known to strongly influence environmental conditions in southeastern Australia [45]. Sustained positive values of SOI (higher than +8) and negative values (lower than −8) often indicate La Niña and El Niño episodes, respectively. Monthly means of SOI were obtained from the Australian Bureau of Meteorology (www.bom.gov.au/climate/current/soihtm1.shtml). In addition, daily sea ice satellite-based observations (resolution: 25 km) were obtained from the National Snow and Ice Data Center (http://neo.sci.gsfc.nasa.gov/view.php?datasetId=NISE_D). Data were extracted between 80°E and 170°E and converted to weekly means during chick-rearing for each study period (2012–2014).

### Data processing and analysis

To investigate habitat use, GPS tracks were analysed with the *trip*, *adehabitatHR* and *adehabitatLT* packages [46, 47] within the R statistical environment (www.R-project.org). An iterative forward/backward speed filter was used to remove unrealistic locations yielding unrealistic high travel speeds [48] (speed threshold >60 km · h^−1^ [29]). On average, the speed filtering removed 0.5 % (range: 0–6.2 %) of recorded locations during individual foraging trips. Analyses were performed on complete foraging trips, defined as the time between when individuals departed from, and when they returned to, the colony. For each foraging trip: (1) trip duration (h); (2) total distance travelled (km); (3) maximum distance to the colony (km); and (4) average bearing (°) from the colony were calculated. To identify the feeding areas of individuals, kernel density estimates (KDE) [49] of GPS locations were generated with grid cells of 0.01°. Because the number of locations recorded was different for each individual, KDE was estimated for each individual and then averaged across individuals to estimate a mean KDE for each colony (see [50]). The smoothing parameter *h* was estimated by least-squares cross-validation in both cases. The 50 % kernel utilization distribution (KUD) was considered to represent the core foraging area and the 95 % KUD the home range of instrumented individuals.

Geolocator loggers recorded light intensity over time, from which geographical positions were then estimated (for details see [51]) with previous studies reporting location accuracy of 185 ± 115 km [51] and 202 ± 171 km [52] for a flying seabird. In brief, the LAT2900 data loggers recorded light intensity every 2 min, processed the light data on-board with proprietary algorithms from the manufacturer (“template fit method”, see [53]), and recorded the estimated daily longitude and latitude. They also recorded environmental temperatures (0.05 °C) and salt-water immersion at 2 min interval. The Mk3005 units measured the light level every minute, recording the maximum reading each 5 min intervals, and also recorded time when salt-water immersion occurred (state changed from wet to dry) and sea surface temperature was recorded after 20 min in the wet phase. The at-sea locations of individuals were then estimated using the *tripEstimation* package [54, 55]. Location estimations calculated from light elevation were constrained by temperature and land masks in order to exclude unrealistic locations [55]. Tracks with two locations per day were obtained. Then a Bayesian state-space model constrained by the average flight speed of adult short-tailed shearwater (30.8 km · h^−1^, [32]) was used to improve the spatial likelihood of the tracks and resample locations at regular 6 h time intervals (R package *bsam*, [56]).

Inter-annual variation in foraging distribution in the Southern Ocean was investigated using kernel analysis on predicted locations with a smoothing parameter *h* of 1.8° (corresponding to a search radius of ~200 km, [57]) and a grid cell size of 0.2°. In order to focus on foraging distribution in the Southern Ocean, locations corresponding to sea-surface temperatures greater than 15 °C were excluded from further analysis (i.e. to remove locations close to the breeding colonies and along the Australian continental shelf). For each year, individuals from both breeding colonies were pooled and differences in distribution between years were based on the proportional overlap of the 50 and 95 % kernels.

In order to investigate short-term response to environmental variability, inter-annual differences in reproductive parameters were tested with a *Chi*-square test. One-way ANOVAs (or circular ANOVA for angular data) followed by Tukeys post hoc tests were used to assess inter-annual variations in foraging parameters and environmental parameters. Dependent variables were log-transformed when necessary in order to meet normality. All analyses were conducted within the R statistical environment [58]. Unless stated otherwise, data are presented as Means ± SD and significance level was set at α = 0.05.

## Results

Some individuals could not be recaptured upon first return to the colony, either due to the friable nature of the nesting habitat preventing access to the studied burrows or because some individuals succeeded in burrowing out after they had fed the chick. As a consequence, few GPS data loggers could be recovered. Other individuals were recaptured after several short foraging trips but by this stage, the GPS data logger had fallen off. Consequently, only 12, 8 and 9 individuals equipped with GPS data logger at Gabo Island and 9, 2 and 10 at Griffith Island were retrieved in 2012, 2013 and 2014, respectively. Furthermore, due to data logger failure, data could not be downloaded for all the loggers retrieved. Complete short foraging trips were obtained from a total of 41 individuals (see Table 1 for details). Due to breeding failure at Griffith Island in 2012, only one individual returned with its GPS data logger and the data, while presented in summary, were excluded from further statistical analyses. While foraging in neritic waters, significant inter-annual differences in at sea movements were only found at Griffith Island. Total distance travelled and maximum distance from the colony were greater in 2014 than in 2012 (*F*_1,17_ = 8.93, *P* = 0.008 and *F*_1,17_ = 10.34, *P* = 0.005, respectively, Table 1). These inter-annual differences in foraging behaviour were associated with differences observed in environmental parameters between the breeding seasons. At Griffith Island, average SST was found to vary between years with higher SST in 2013 (*F*_2,42_ = 10.45, *P* < 0.001, Table 2). On the other hand, average SST at Gabo Island was higher in 2014 (*F*_2,44_ = 12.37, *P* < 0.001). At a meso-scale, the strength of the Bonney Upwelling was found to be the weakest during the austral summer 2013, resulting in significantly higher SST (*F*_2,32_ = 13.31, *p* < 0.001).

**Table 1:** Inter-annual comparison of mean (± SD) short foraging trip parameters in short-tailed shearwaters rearing chicks at two breeding colonies

Colony	Years	2012	2013	2014	*F*	*df*	*P*-value
Gabo Island	Birds tracked	9	8	4			
	Trip duration (h)	16.2 ± 1.1	16.2 ± 1.4	17.2 ± 3.2	1.29	2, 18	0.30
	Total distance travelled (km)	188.8 ± 58.6	182.1 ± 72.5	159.5 ± 51.7	2.27	2, 18	0.13
	Maximum distance (km)	41.2 ± 8.6	41.9 ± 18.0	26.0 ± 9.1	0.15	2, 18	0.86
	Bearing (°)	138.5 ± 109.9	91.5 ± 102.3	202.3 ± 38.7	2.21	2, 18	0.14
	Home range (km^2^)	663.3 ± 217.8	827.1 ± 379.2	561.9 ± 214.3	1.28	2, 18	0.30
	Foraging area (km^2^)	124.0 ± 33.2	179.0 ± 86.0	118.1 ± 69.3	1.93	2, 18	0.17
Griffith Island	Birds tracked	9	1^a^	10			
	Trip duration (h)	18.9 ± 7.1	18.2	25.3 ± 11.4	2.06	1, 17	0.17
	Total distance travelled (km)	269.2 ± 143.1	276.0	581.3 ± 222.4	**8.93**	**1, 17**	**0.008**
	Maximum distance (km)	78.3 ± 35.0	107.2	156.6 ± 54.4	**10.34**	**1, 17**	**0.005**
	Bearing (°)	148.6 ± 54.4	115.9	158.9 ± 63.2	0.11	1, 17	0.74
	Home range (km^2^)	1301.7 ± 687.7	1180.9	3271.1 ± 1406.1	**14.48**	**1, 17**	**0.001**
	Foraging area (km^2^)	193.6 ± 124.6	182.3	606.2 ± 288.9	**15.66**	**1, 17**	**0.001**

Significant results are indicated in bold (*P* > 0.05)

^a^Results for Griffith Island in 2013 were not included in the analysis

**Table 2:** Inter-annual comparison of mean (± SD) environmental parameters for the short-tailed shearwater chick-rearing period (January–April)

Local conditions		2012	2013	2014	*F*	*df*	*P*-value
Gabo Island	SST (°C)	19.7 ± 0.8^*^	20.2 ± 0.9^*^	21.2 ± 0.8^**^	**12.37**	**2, 44**	**<0.001**
	Chl-*a* (mg.m^−3^)	0.5 ± 1.0	0.5 ± 0.6	1.1 ± 1.1	2.55	2, 44	0.09
	Wind speed (m.s^−1^)	8.6 ± 2.2	8.4 ± 2.0	9.0 ± 2.4	1.01	2, 358	0.36
	Wind direction (°)	138.7 ± 47.9	138.9 ± 48.7	132.2 ± 55.9	0.86	2, 358	0.43
Griffith Island	SST (°C)	18.0 ± 0.6^*^	18.7 ± 0.5^**^	17.6 ± 0.5^*^	**10.45**	**2, 42**	**<0.001**
	Chl-*a* (mg.m^−3^)	0.2 ± 0.2	0.2 ± 0.2	0.2 ± 1.1	1.58	2, 42	0.22
	Wind speed (m.s^−1^)	7.8 ± 1.8	7.1 ± 1.7	7.8 ± 1.7	0.64	2, 358	0.53
	Wind direction (°)	156.5 ± 52.0	173.4 ± 58.6	179.1 ± 43.4	1.30	2, 358	0.27
Meso and large-scale processes							
Bonney Upwelling SST (°C)		17.6 ± 0.7^*^	18.8 ± 0.5^**^	17.6 ± 0.6^*^	**13.31**	**2, 32**	**<0.001**
SOI		−0.8 ± 5.7	4.0 ± 5.8	−2.1 ± 8.4	2.67	2, 31	0.09
Sea ice concentration (%)		57.2 ± 27.4^*^	70.5 ± 26.5^**^	59.6 ± 27.7^*^	**31.04**	**2, 47**	**<0.001**

Local conditions included sea surface temperature (*SST*), sea surface chlorophyll-*a* concentration (*Chl*-*a*), wind speed and wind direction. Meso-scale process included Bonney Upwelling sea surface temperatures (*SST*) over the austral summer (January–March) and large-scale processes included the Southern Oscillation Index (*SOI*) over a yearly period and the sea ice concentration in the Antarctic region. Significant results are indicated in bold and homogenous subsets (*P* > 0.05) are indicated by asterisks

**Fig. 2:**
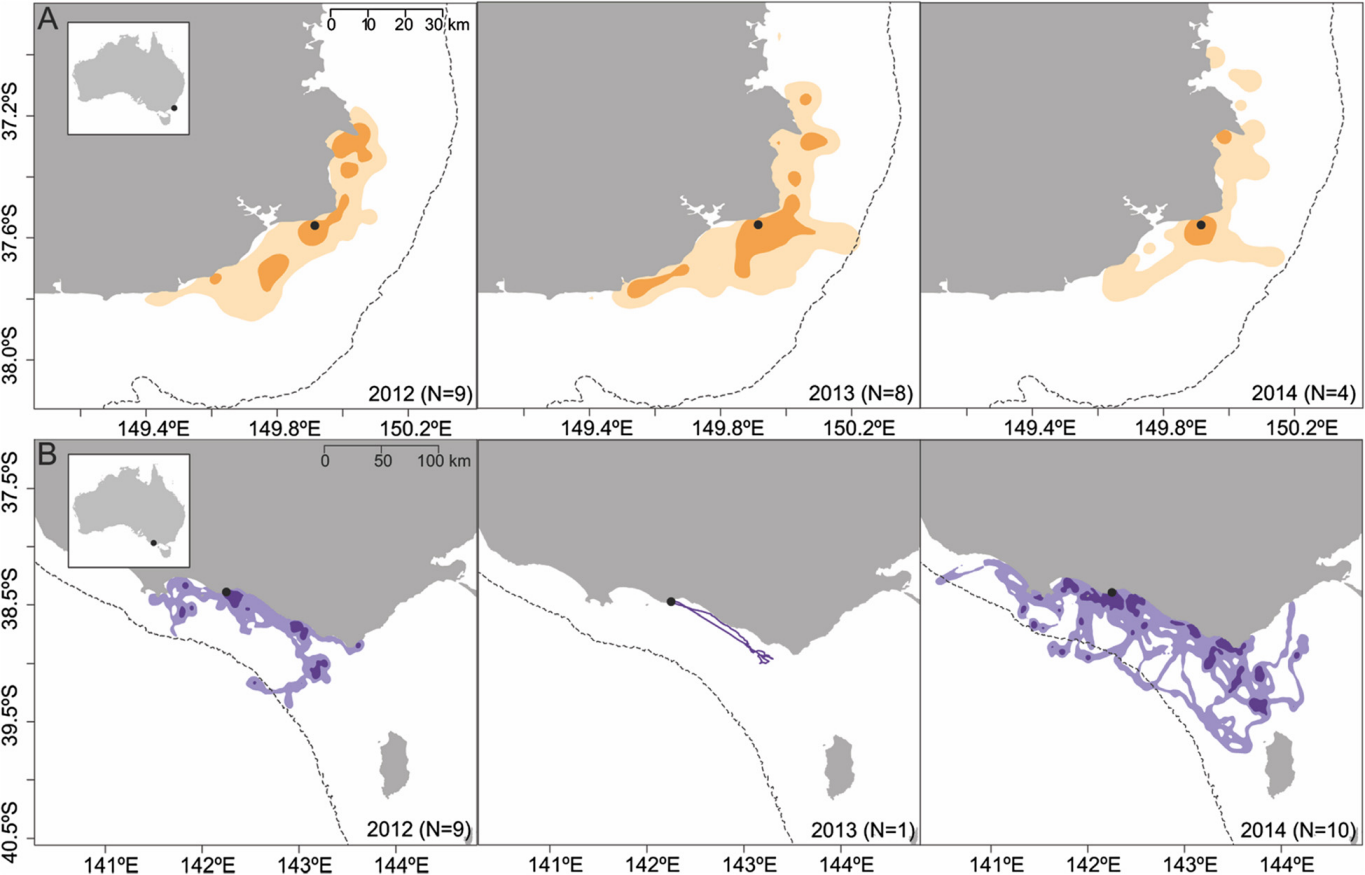
Distribution of short-tailed shearwaters foraging in Bass Strait. Results of a kernel density estimate analysis for short-tailed shearwaters foraging from (**a**) Gabo Island and (**b**) Griffith Island breeding colonies in 2012, 2013 and 2014 (data presented for Griffith Island in 2013 are strictly informative). *Darker shade* colors represent the core foraging area (50 % KUD contour), while *lighter shade* colors represent the home range (95 % KUD contour). The *dashed line* indicates the location of the 200 m isobath. *Inset* map shows the colonies’ location in relation to Australia

At Gabo Island, the short foraging trip hot spots during the study period were located close to the colony in inshore areas. Birds from Griffith Island foraged both inshore and over the shelf-edge, extending their home range towards King Island in western Bass Strait in 2014 (Fig. 2a and b). At Griffith Island, core foraging and home range areas were significantly greater in 2014 than in 2012 (*F*_1,17_ = 15.66 and *F*_1,17_ = 14.48 respectively, *P* = 0.001 in both cases).

Due to logistical constraints (i.e. limited period of time to recapture individuals instrumented with geolocators at their breeding sites), some individuals could not be recaptured. Consequently, only 5, 3 and 13 individuals equipped with geolocators at Gabo Island and 6, 3 and 12 at Griffith Island were recaptured in March). Data logger failure prevented data to be downloaded from several geolocators retrieved. Complete long foraging trips were obtained from a total of 35 individuals. When multiple trips were recorded, only the first long trip was kept for further analysis. In addition, due to the small number of birds recovered with GLS data logger during the study period, data from both colonies were pooled together for inter-annual comparisons. Long foraging trips duration ranged from 16.4 ± 2.6 days in 2012 to 19.8 ± 6.3 days in 2014 (2013: 17.1 ± 2.2 days). The total distance travelled ranged from 10,364 ± 1958 km in 2012 to 11,260 ± 2965 km in 2014 (2013: 12,345 ± 3754 km). A significant inter-annual difference in long foraging trip parameters was found only in maximum distance travelled from the colony, which was greater in 2014 (4419 ± 1010 km) than in 2012 (3393 ± 376 km) and 2013 (4074 ± 751 km) (*F*_2,32_ = 4.48, *P* = 0.02).

**Fig. 3:**
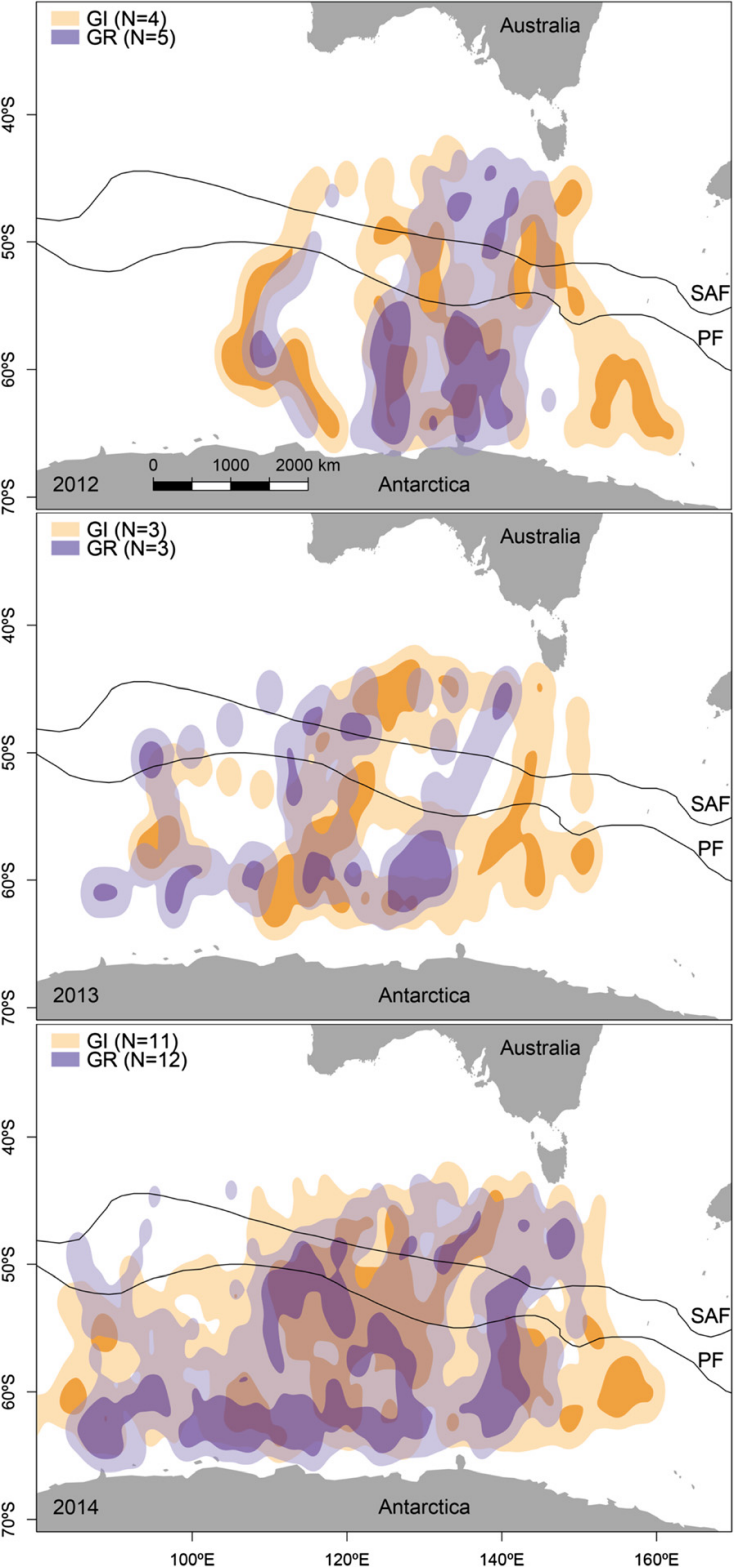
Distribution of short-tailed shearwaters foraging in the Southern Ocean. Results of a kernel density estimate analysis for GLS-tracked short-tailed shearwaters from Gabo Island (*GI*) and Griffith Island (*GR*), performing long foraging trips during the chick-rearing period in 2012, 2013 and 2014. The *darker orange* (*GI*) and the *darker purple* (*GR*) colors represent the core foraging area (50 % KUD contour), while the *lighter orange* and the *lighter purple* colors represent the home range (95 % KUD contour). Oceanic frontal zones: sub-Antarctic waters between the Sub-Antarctic Front (*SAF*) and the Polar Front (*PF*), and Antarctic waters south of the PF (from [82, 83])

In 2012, individuals exploited mainly the oceanic zone located south of the Polar Front (PF), as well as the Sub-Antarctic waters located between the Sub-Antarctic Front (SAF) and the PF (Fig. 3). In 2013, individuals foraged principally in the Antarctic region, as well as the oceanic area located north of the SAF. In 2014, individuals from both breeding sites exploited Sub-Antarctic waters, as well as Antarctic waters and areas located north of the SAF and the areas exploited were more widespread longitudinally. Core foraging areas overlapped by 18 % between 2012 and 2013, 27 % between 2012 and 2014, and 34 % between 2013 and 2014. Home range areas overlapped by 58 % between 2012 and 2013, 71 % between 2012 and 2014, and 79 % between 2013 and 2014.

Significant inter-annual differences in reproductive performances were found at both colonies. At Gabo Island, hatching success was lower in 2013 (*χ*^*2*^ = 9.82, *P* < 0.001, Table 3). Additionally, fledging and breeding success was lower in 2013 than in 2014, and lower in 2013 than in 2012 (*χ*^*2*^ = 22.46, *P* < 0.001). Similar trend was found at Griffith Island for hatching success (i.e. lowest value in 2013, *χ*^*2*^ = 15.08, *P* < 0.001) whereas fledging and breeding success was lower in 2013 than in 2012 and 2014 (*χ*^*2*^ = 9.99, *P* = 0.007).

**Table 3:** Inter-annual comparison of mean (± SD) reproductive parameters of short-tailed shearwaters

Colony	Study year	2012	2013	2014	*F*	*df*	*P*-value
Gabo Island	Nests	50	70	81			
	Hatching success (%)	80.0^*^	52.9^**^	70.4^*^	**9.82**	**2**	**<0.001**
	Fledging success (%)	55.0^*^	13.5^**^	31.6^***^	**22.46**	**2**	**<0.001**
	Breeding success (%)	44.0^*^	7.1^**^	22.2^***^	**22.45**	**2**	**<0.001**
Griffith Island	Nests	106	109	97			
	Hatching success (%)	65.1^*^	39.4^**^	57.7^*^	**15.08**	**2**	**<0.001**
	Fledging success (%)	50.7^*^	37.2^**^	41.1^*,**^	**9.98**	**2**	**0.006**
	Breeding success (%)	33.0^*^	14.7^**^	23.7^*,**^	**9.99**	**2**	**0.007**

Parameters were recorded over three consecutive breeding seasons. Nests is the total number of nests monitored each year. The number of adults measured to calculate body condition index is indicated in brackets. Significant results are indicated in bold and homogeneous subsets (*P* > 0.05) are indicated by asterisks

## Discussion

Consistent with previous observations [27, 29], the results of the present study indicate short-tailed shearwaters performed daily short foraging trips over the study period (with the exception of 4 individuals in 2014 from Griffith Island that performed 2-day trips). At Gabo Island, short foraging trips were exclusively performed in inshore areas 20–80 km around the breeding colony. However, total distance travelled and foraging range did not vary between 2012 and 2014, despite higher SST recorded during summer 2014. In contrast, at Griffith Island, foraging occurred both in inshore habitat and over the continental shelf-edge (20–240 km from the colony). Inter-annual variations were found in short foraging trip parameters but those differences were mainly due to the individuals performing longer foraging trips in 2014 and, thus, were able to extend their range further away from the colony. While data was retrieved from only a single individual in 2013, the location of its foraging areas is consistent with those in the two other years.

Despite the small sample size, this study showed that while performing long foraging trips, birds from both breeding colonies converged on the same areas in the Southern Ocean. Individuals travelled 2400–6100 km across oceanic regions, repeatedly using sub-Antarctic and Antarctic Zones between longitude 80 and 160° across the study period. These results are consistent with previous studies [33, 34] and likely reflect enhanced primary productivity and a high prey availability associated with these regions, where shearwaters mainly feed on Antarctic krill and myctophid fish [28, 31]. Longitudinal range was narrower in 2012 than in 2013 and 2014 and, while non-significant, there was a trend for the average total distance travelled in the Southern Ocean region and the average maximum distance travelled from the colonies to increase between 2012 and 2014 for both colonies.

Therefore, the results of the present study are inconsistent with the prediction that, during years of lower resource availability, short-tailed shearwaters should further increase their range when performing short foraging trips. However, the results suggest that they are able to do so while exploiting distant feeding zones. Substantial inter-annual variability has also been shown among other species, with individuals being able to modulate their dual foraging strategy according to food availability [22, 59–61]. This could indicate that individuals performing longer foraging trips during years of poor conditions use this strategy not only to restore their body reserves but also to maximise foraging efficiency by reducing energetic costs. Longer foraging trips to distant, yet more predictable and profitable areas are likely to be more energetically efficient than exploiting smaller patches with lower marine productivity around the colony [16, 22].

Exploiting resources over a broad area has been interpreted as a mechanism of regulating investment in offspring [18, 31] and, therefore, breeding success and adult survival are closely associated to changes occurring in the feeding grounds. Links between chick growth and adult mass mortalities, and fluctuations in resource abundance were previously reported [62]. Dramatically low reproductive success was recorded for both colonies in 2013. Griffith Island is located close to the Bonney Upwelling and the waters around it are under its direct influence. The low breeding success observed in 2013 is consistent with the low strength of the Bonney Upwelling that year (as indicated by higher SST) which could have reduced prey availability for shearwaters. Indeed, lower breeding performances were also observed for little penguins *Eudyptula minor* (Berlincourt et al. unpublished data), Australasian gannets *Morus serrator* (Angel et al. unpublished data) and Australian fur seals *Arctocephalus pusillus doriferus* (Arnould et al. unpublished data) breeding throughout Bass Strait in the same year suggesting unusually low prey availability for these predators in the region. The low breeding success observed at Gabo Island at the eastern extent of Bass Strait could also be related to the consequences of a weaker Bonney Upwelling throughout the region [63], or could be linked to pulses of warmer water being brought by the southward penetration of the EAC [64] impacting prey distribution [65].

The breeding season 2014 was characterized by a lower breeding participation (i.e. lower number of adults incubating in November 2013, pers. data), after mass adult mortality occurred in September 2013 (pers. data) when adults returned from their trans-equatorial migration [66]. Severe weather and difficulty finding sufficient resources are likely to have contributed to this event. However, despite the possible reduction of density dependent competition for food resources, low reproductive performance was also recorded in 2014, with a lower breeding success recorded in 2014 than in 2012 (implying that oceanographic conditions were still not optimum). This result could be explain by a cascade of events following the weak Bonney Upwelling observed during summer 2013 and leading to lower nutrient concentration in Bass Strait.

Importantly, while individuals from both colonies did not appear to increase their effort when foraging locally during the periods of reduced local prey availability (inferred from the lower fledging success), our results suggest that they increased their foraging range in the Southern Ocean. This could indicate that they were buffering variability in local resource availability by exploring and exploiting Antarctic waters more extensively. Indeed, when performing long trips, short-tailed shearwaters mainly forage south of the Polar Front zone in waters where they are known to feed on Myctophid fish and Antarctic krill (*Euphasia superba*), which are abundant in this region [67–69]. Around Antarctica, the pack-ice provides a nursery ground for Antarctic krill larvae over the winter months. Over summer, the decay of the sea-ice is the most important physical process and has a large impact on the marine fauna foraging in this region; as the sea-ice retreats abundant resources become accessible to predators [67, 70, 71].

During summer 2013, sea-ice extent was greater around Antarctica (as indicated by higher sea-ice concentration), which could have lead to less resource available for the shearwaters. In fact, the seasonal increase in krill abundance has been shown to be associated with the spring/summer retreat of sea-ice (i.e. sea-ice is progressively broken into floes). As summer progresses, sea-ice extent decreases and resources become available for seabirds [72, 73]. Therefore, concentration of krill will depend on the extent and duration of sea-ice cover [74]. Shearwaters might have had to extend their long foraging trips while searching for food in the Southern Ocean because of poorer conditions in this region in 2013. This, in conjunction with very poor environmental conditions in Bass Strait could have resulted in very low reproductive success the same year. However, over the study period, longer foraging trips were observed in 2014 suggesting that individuals had to extend their foraging range because oceanographic conditions either around their respective breeding colony or in the Southern Ocean were not optimum.

## Conclusions

In summary, this study has highlighted how long foraging trips in short-tailed shearwaters may serve as a buffer against local environmental variations to maintain and/or replenish adult body condition, and also feed chicks throughout chick rearing. Flexibility in foraging strategy allows the birds to travel southward where they can exploit extensive cold, productive Antarctic waters and enabling them to cope with variability in local prey availability around the breeding colony during chick-rearing. The oceanic region of south-eastern Australia is one of the fastest warming marine areas in the world [75, 76] and is likely to experience substantial alterations to the local oceanography and species distributions [64]. The complex oceanographic processes around Australia (e.g. EAC, Leeuwin current, Bonney Upwelling) have also been predicted to alter with anticipated climate change [77, 78]. Furthermore, shifts in the Antarctic marine food web have also been reported [79]. Such changes are likely to impact the prey distribution [65, 69, 80], foraging success, chick growth and, ultimately, reproductive success of short-tailed shearwaters. Furthermore, despite less favourable conditions, short-tailed shearwaters are more likely to invest in breeding season than abandon the breeding attempt prior to egg laying. This suggests that individuals of higher quality could be able to breed more frequently than others without compensatory reduction in their reproductive success or their chance of future survival [81].
